# Reduced irradiation exposure areas enhanced anti-tumor effect by inducing DNA damage and preserving lymphocytes

**DOI:** 10.1186/s10020-024-01037-w

**Published:** 2024-12-31

**Authors:** Huiqin Chen, Yuan Li, Qiaofeng Shen, Guanqun Guo, Zhigang Wang, Hanyu Pan, Min Wu, Xueqing Yan, Gen Yang

**Affiliations:** 1 Wenzhou Institute, University of Chinese Academy of Sciences, Wenzhou, 325000 China; 2 State Key Laboratory of Nuclear Physics and Technology, School of Physics, Peking University, Beijing, 100871 China; 3 School of Public Health, Wenzhou Medical University, Wenzhou, 325035 China; 4 South Zhejiang Institute of Radiation Medicine and Nuclear Technology, Wenzhou, 325014 China; 5 Postgraduate Training Base Alliance of Wenzhou Medical University, Wenzhou, 325035 China

**Keywords:** Radiotherapy, Target areas, DNA damage, Immune activation

## Abstract

**Background:**

Partial stereotactic body radiation therapy (SBRT) targeting hypoxic regions of large tumors (SBRT-PATHY) has been shown to enhance the efficacy of tumor radiotherapy by harnessing the radiation-induced immune response. This approach suggests that reducing the irradiation target volume not only achieves effective anti-tumor effects but also minimizes damage to surrounding normal tissues. In this study, we evaluated the antitumor efficacy of reduced-tumour-area radiotherapy (RTRT) , and explored the relationship between tumor control and immune preservation and the molecular mechanisms underlying of them.

**Methods:**

In mouse breast cancer models, we compared the anti-tumor effects of RTRT and conventional radiotherapy (CNRT) by assessing tumor growth, metastasis, and survival rates. Additionally, we evaluated the peritumoral tissue damage and the immune microenvironment. The maturation of dendritic cells (DCs) and DNA damage induced by irradiated tumor cells were also assessed in vitro.

**Results:**

In pre-clinical models, both RTRT and CNRT significantly inhibited primary tumor growth when compared to non-irradiated controls, with no significant difference between RTRT and CNRT. However, RTRT significantly extended survival times in mice, and increased the likelihood of inducing abscopal effects, thereby providing potential for better control of distant metastases. Further investigations revealed that the enhanced efficacy of RTRT may be attributed to the preservation of lymphocytes within the peritumoral tissue, as well as reduced damage to the surrounding skin and circulating lymphocytes. In vitro assays demonstrated that RTRT induced DNA damage and dsDNA in tumor cells, activating the cGAS-STING pathway. RTRT also triggered the release of damage-associated molecular patterns (DAMPs), which synergistically amplified the anti-tumor immune response.

**Conclusions:**

Our findings suggested that appropriately narrowing the irradiation target volume effectively killed tumor cells while reducing damage to surrounding tissues, and preserving peritumoral lymphocytes. This approach improved the safety of radiotherapy while maintaining its efficacy in tumor control and provided an opportunity for combining high-dose radiotherapy with immunotherapy.

**Supplementary Information:**

The online version contains supplementary material available at 10.1186/s10020-024-01037-w.

## Introduction

Radiotherapy (RT), together with surgery and chemotherapy, constitutes the “troika” of contemporary cancer treatment modalities. It is estimated that about half of cancer patients receive RT at some stages, with indications ranging from curative intent to symptom relief (Barton et al. 2014; Citrin 2017). RT is also highly cost-effective, accounting for only 5% of overall cancer care costs, compared to 50% for surgery (De Ruysscher et al. 2019). By inducing catastrophic DNA damage, RT leaded to tumor regression (Huang and Zhou 2020).

Advancements in science and technology have significantly enhanced the efficacy of tumor radiotherapy, enabling precise delivery of radiation doses to tumors while minimizing exposure to surrounding normal tissues (Barazzuol et al. 2020). Current research efforts are focused on improving the accuracy of radiotherapy through image-guided techniques, thereby reducing the radiation dose to normal tissues and minimizing adverse effects. One such technique, intensity-modulated radiotherapy (IMRT), enables the delivery of high doses to the tumor while limiting the exposure of normal tissues to high radiation doses, although it may result in an increased exposure of surrounding tissues to lower doses (De Ruysscher et al. 2019; Barazzuol et al. 2020). Another approach, volumetric modulated arc therapy (VMAT), utilizes rotational irradiation in a 360-degree multi-arc configuration, allowing for more comprehensive targeting of the tumor volume. This technique not only reduces the high-dose exposure to adjacent normal tissues but also improves the uniformity of dose distribution within the tumor target (Alsaihaty et al. 2024). However, VMAT requires advanced equipment and highly skilled personnel to ensure both the precision and safety of the treatment.

Radiotherapy also affects surrounding normal tissues, often causing lymphopenia by direct or indirect effects, especially in sensitive lymphocytes, which can negatively impact tumor control. To overcome the radio-resistance of tumor cells, it is often necessary to increase the radiation dose to enhance the therapeutic efficacy. However, conventional radiotherapy (CNRT), which targets the entire tumor tissue along with surrounding normal tissues (Zietman et al. 2005), inadvertently damage healthy tissues. This includes radiation-sensitive immune cells within the tumor, which may lead to lymphopenia. Such damage can disrupt the tumor immune microenvironment, thereby diminishing the overall therapeutic response (Filatenkov et al. 2015; Basler et al. 2018; Morisada et al. 2018). Moreover, the radiation tolerance of normal tissues limits the potential for further dose escalation, posing a significant challenge to improving the efficacy of radiotherapy (Anon 2022; Hong et al. 2024). These considerations underscore the critical need to explore the balance between effective tumor control and the protection of normal tissues in radiation oncology.

Onishi et al. demonstrated that CNRT, like stereotactic body radiotherapy (SBRT), provides improved local control and survival benefit when the dose exceeding 100 Gy. However, a notable downside is the increased risk of radiation toxicity when the irradiated target area expands (> 5–7 cm) or approaches critical normal tissues (Onishi et al. 2007; Allibhai et al. 2013; Kang et al. 2015). The risk of side effects is closely tied to both the radiation dose and the radiation area of normal tissue. It is important to highlight that reducing the irradiation target areas in existing radiotherapy protocols does not compromise therapeutic efficacy but can enhance safety. For example, in breast cancer patients, limited-field radiotherapy yields similar 5-years local control rates to whole breast radiotherapy while reducing the risk of contralateral breast complications (1% vs 4%) (Vicini et al. 2003). Furthermore, Conway and colleagues found that smaller field radiation therapy carried less than half the risk of secondary breast cancer compared to mantle field radiation therapy, with no increased risk when combined with chemotherapy (Conway et al. 2017). The concept of SBRT-PArtial Tumor Irradiation Targeting HYpoxic Segment (SBRT-PATHY) developed by Tubin et al. (2020), is noteworthy for its ability to preserve the immune environment surrounding the tumor. This approach takes advantage of the bystander and abscopal effects of radiation, leveraging the immune system to elicit a more robust anti-tumor response. Despite these promising findings, there remains a scarcity of studies investigating for partial tumor irradiation.

Based on these findings, we investigated the relationship between radiotherapy-induced tumor control and immune protection mediated by reducing tumor area radiation (RTRT), and explored the molecular mechanisms underlying its anti-tumor effects. Research has shown that approximately 40% RTRT achieved tumor control comparable to CNRT, while reducing normal tissue damage and enhancing the safety of tumor radiotherapy. This effect is mediated through the activation of the cGAS-STING pathway, triggered by RTRT-induced dsDNA and DAMPs, which in turn initiate an anti-tumor immune response. Furthermore, the preservation of peritumoral immune cells and circulating lymphocytes by RTRT enhances the likelihood of exploiting abscopal effects, enabling systemic control of non-irradiated tumors. These results highlight the potential benefits of reducing radiation fields in clinical RT to improve safety and efficacy.

## Materials and methods

### Mice

BALB/c wild-type mice were purchased from Experimental Animal Center of Zhejiang Province and housed in the animal facility of the Wenzhou Institute, University of Chinese Academy of Sciences. All animal experiments were reviewed and approved by the Institutional Animal Care and Use Committee of Wenzhou Institute, University of Chinese Academy of Sciences.

### Cell lines and irradiation

Mouse breast cancer 4T1-Luc cells were cultured in RPMI-1640 medium supplemented with 10% fetal bovine serum (FBS) and 1% penicillin/streptomycin. Cells maintained at 37 °C in a humidified atmosphere with 5% CO_2_. The 4T1-Luc cells were a gift from Wenzhou Medical University. For 100% irradiation (IR-100%), 4T1-Luc cells were irradiated with 12 Gy (6 Gy/min) of X-rays. For partial irradiation, tumor cells with 10%, 40% or 50% of the bottom area of the culture vessel was exposed to 12 Gy of X-rays at a dose rate of 6 Gy/min.

### Dendritic cell activation

Bone marrow-derived dendritic cells (BMDCs) were isolated from the femurs and plated in culture dishes in RPMI-1640 medium supplemented with 10% fetal bovine serum (FBS) and 1 μg/mL GM-CSF (CK02, novoprotein) and 1 μ/ml IL-12 (CK74, novoprotein). The cell culture media were replenished on days 3, 4, and 6. After 7 days of differentiation, BMDCs were harvested for subsequent experiments. To observe BMDC activation by irradiated tumor cells, BMDCs were incubated for 24 h with different proportions of irradiated tumor cells. Lipopolysaccharide (LPS) at a final concentration of 1 μg/mL as a positive control. Following co-culture, cell mixture were harvested and stained with antibodies for 30 min at 4 °C. The percentages of mature BMDC were detected by flow cytometry with CD86^+^/CD80^+^ cells. The following antibodies were used: CD45-AF700 (560510, BD), CD11c-BV421 (117329, Biolegend), CD80-APC (104714, Biolegend) and CD86-PE (159204, Biolegend).

### DAMPs determination

The release of damage-associated molecular patterns (DAMPs) from irradiation tumor cells were assessed by measuring the secretion of heat shock proteins 70 (HSP70) and heat shock proteins 90 (HSP90). The secretion levels of HSP70 (A23013068, CUSABIO) and HSP90 (A22013067, CUSABIO) were quantified using commercially available ELISA kit according to the manufacturers instructions. Measured concentrations were normalized to the number of viable cells.

### Dose stability test for small target volume irradiation

The stability of dose delivery to small target volumes was assessed using EBT3 Gafchromic film (0810, Gafchromic, USA). The film was placed at the location corresponding to the irradiation site of the mice. The irradiation was performed with three different dose levels (12 Gy, 15 Gy, and 18 Gy) using two field sizes 0.5 cm * 0.5 cm and 1 cm * 1 cm. Each dose was repeated three times. Following irradiation, the images were quantitative analysis using Image J software (Version 1.8.0.112, National Institutes of Health, MD, USA).

### Tumor models and treatment

1. The unilateral therapeutic model: Five-week-old BALB/c mice were randomly divided into three groups and subcutaneously injected with 4T1-Luc cells (1 × 10^6^ cells in 100 μL of normal saline per mouse) into the right flank. On day 8, when the tumor areas reached 200–250 mm^3^, tumros were irradiated with a single dose of 15 Gy delivered at a dose rate of 6 Gy/min. Tumor irradiation was performed as follows: in 40% partial tumor irradiation group (RTRT), the irradiated target areas covered 40% of the maximum tumor area (length × width). In the conventional irradiation group (CNRT), the irradiated target areas was ≥ 95% of the maximum tumor area.
2. Immune cells depletion model: For immune cell depletion, 5-week-old BALB/c mice were randomly assigned to various experimental groups and subcutaneously injected with 4T1-Luc cells (1 × 10^6^ cells in 100 μL of normal saline per mouse) in the right flank. To deplete specific immune cells, anti-mouse CD4^+^ T (BE0003, Biolegend) /CD8^+^ T (BE0223, Biolegend) /NK cells mAbs (BE0036, Biolegend) or its isotype control mAbs were administered intraperitoneally at a dose of 200 μg per mouse on days 9, 11, 13 and 15. On day 10, when the tumor volume reached approximately 200 mm^3^, tumors were irradiated with RTRT or CNRT as described above.
3. Bilateral therapeutic model. In the bilateral therapeutic model, 5-week-old BALB/c mice were randomly divided into each group and first subcutaneously injected with 4T1-Luc cells (1 × 10^6^ cells in 100 μL of normal saline per mouse) in the right flank. On day 9, a second injection of 4T1-Luc cells (2 × 10^6^ cells in 100 μL of normal saline per mouse) to the left flank of the same animals. Mice were randomly divided into three groups. On day 13, when the right tumor volume reached 300–400 mm^3^, the tumor was irradiated with RTRT or CNRT as described above.

### Tumor size measurement

Tumor growth in all models was monitored by measuring tumor dimensions using a digital caliper. Tumor volume was calculated using the following ellipsoidal formula:

$${\text{V}} = {1}/{2} \times \left( {{\text{length}}} \right) \times \left( {{\text{width}}} \right)^{{2}} .$$

### Detection of lung metastasis

Mouse lung tissues were collected at the end of the experiment, washed with PBS, and fixed with tissue fixative for at least 24 h. After fixation, the fixative was carefully removed using absorbent paper. The number of tumor metastases on both the front and back sides of the lung tissue was then observed and counted.

### Flow cytometric analyses

For the analysis of tumor-infiltrating lymphocyte, tumor tissues were harvested at the end of the experiment. Cell suspensions were prepared by enzymatic hydrolysis of the tumor tissue. Flow cytometric analyses of tumor-infiltrating lymphocytes were performed using the following antibodies: CD45-AF700 (560510, BD), CD3-FITC (553061, BD), CD8-APC-Cy7 (557654, BD), CD4^+^ Percp-Cy5.5 (550954, BD), and NK1.1-PE (557391, BD). All antibodies were purchased from BD Biosciences.

### Western blot assay

Protein expression was evaluated by Western blot. The total proteins were extracted and separated by SDS-PAGE, then transferred to a PVDF membrane (Millipore, USA). Next, the membranes was blocked with 5% skimmed milk and incubated with the primary antibody overnight at 4 °C. This study employed the following primary antibodies: Anti-GAPDH (A19056, ABclonal), Anti-p-STING (72971, CST), Anti-p-TBK1 (5483, CST), Anti-HSP70 (4872, CST), Anti-HSP90 (ab203085, Abcam), Anti-Three-prime repair exonuclease 1 (TREX1, NBP1-76977, Novus Biologicals), Anti-γH2AX (ab22551, Abcam). After washing with TBST, the membranes were incubated with appropriate HRP-conjugated secondary antibodies for 1 h at room temperature. HRP goat anti-mouse IgG (AS003, ABclonal) or HRP goat anti-rabbit IgG (AS014, ABclonal) was used as a secondary antibody. Protein bands were visualized using an ECL detection assay kit (Amersham Pharmacia Biotech, Buckinghamshire, UK). Quantification of protein bands was performed using Image J software.

### Hematoxylin–eosin (HE) staining

Skin and tumor tissue from different experimental groups were collected and fixed with tissue fixation solution (G1101, Servicebio) at room temperature. After fixation, the tissue were dehydrated by an ethanol gradient, embedded in paraffin, sliced, dehydrated, and hydrated. The sections were then subjected to HE staining. The histopathology of skin and tumor tissue stained with HE was observed under microscope.

### Immunohistochemistry (IHC) analyses

For IHC analysis, paraffin sections were routinely dewaxed and rehydrated. After antigen repair, they were placed in a 3% catalase solution to remove endogenous peroxidase. After washing the sections with PBS (5 min × 3), they were blocked with goat serum for 30 min at room temperature. Sections were then incubated overnight at 4 °C with the following primary antibodies: CD4 (GB15064, Servicebio), CD8 (GB115692, Servicebio) and Ki67 (GB121141, Servicebio). The next day, sections were washed with PBS and incubated with a secondary antibody for 30 min at room temperature. Following PBS washing, the sections visualized using DAB solution, counterstained with hematoxylin, dehydrated through a graded ethanol series, and imaged.

### Statistical analysis

The data are presented as mean ± standard deviation (SD) or standard error of the mean (SEM). Statistical analysis was performed with GraphPad Prism (version 8.3.0 GraphPad Software, La Jolla, CA, USA). Data were analyzed using t-test, one-way or two-way ANOVA. Kaplan–Meier survival curves were generated to evaluate mouse survival, and statistical significance was determined by the log-rank (Mantel-Cox) test. All statistical tests were two-sided. A *P*-value of less than 0.05 was considered statistically significant.

## Results

### Detection of irradiation device and determination of irradiation target areas

**Fig. 1:**
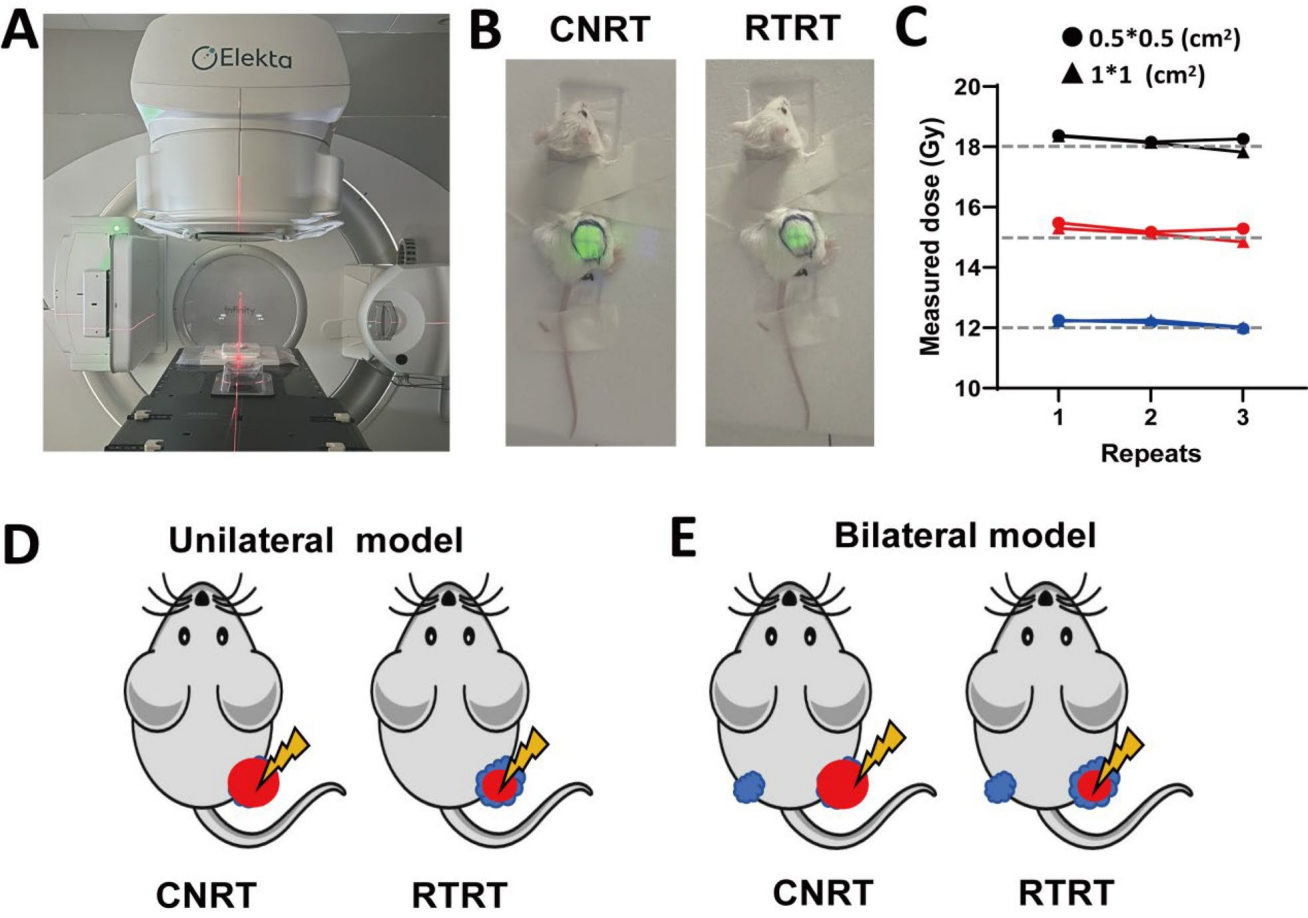
The irradiation device and the target areas for tumor irradiation. **A** Laser location (Red) during irradiation. **B** Simulated photographs of conventional radiotherapy (CNRT) and reduced tumor area radiotherapy (RTRT) of mouse tumors (Green). **C** Stability of irradiation dose for small area irradiation. The gray line represents the theoretical value of irradiation. **D** Schematic of mouse tumor irradiation in a unilateral model. Blue: tumor. Red: irradiated target areas. **E** Schematic of mouse tumor irradiation in a bilateral model. Blue: tumor. Red: irradiated target areas

In this study, a medical linear accelerator (Elekta Infinity, Elekta, Stockholm, Sweden) was utilized for tumor irradiation in mouse model. A red laser was employed for precise localization of the tumor tissue before irradiation (Fig. 1A). Given the significantly small tumor tissue in mice compared to human patients. To ensure precision in small-field irradiation, dosimetric films were used to simulate the irradiation of mouse tumors and to assess the stability of small-area irradiation. Our results showed good dose stability for small fields of 1 * 1 cm^2^ and 0.5 * 0.5 cm^2^ (Fig. 1C). During irradiation, the mice were carefully immobilised, with particular attention to restraining the tumor-bearing region while leaving the head free for natural movement. The tumor was exposed to the irradiation field, and the tumor irradiation boundaries of each tumor were delineated using green fluorescence prior to the treatment (Fig. 1B). The fluorescent-marked region corresponded to the actual irradiation field. In CNRT group, nearly the whole tumor (≥ 95%) was exposed to radiation, while in RTRT, only part of maximum cross-sectional area of the tumor (length × width) were irradiated (Fig. 1B, D, E). In the unilateral model, mice were inoculated with a single tumor (Fig. 1D), wheres in the bilateral model, two tumors were implanted, but only the larger tumor was irradiated (Fig. 1E).

### RTRT shows a similar anti-tumor effect with CNRT

**Fig. 2:**
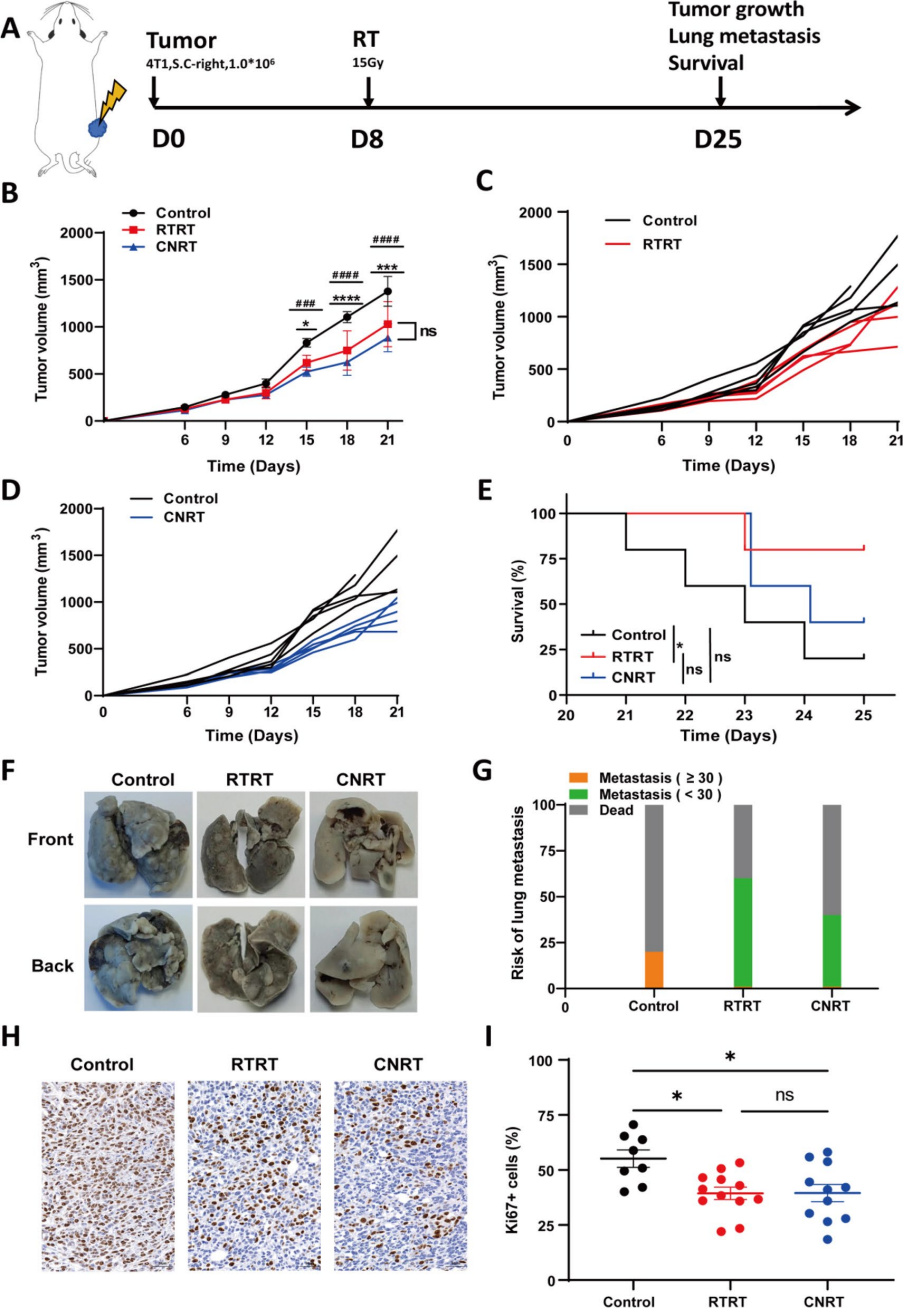
RTRT is comparable to CNRT in tumor control. **A** Schematic diagram of tumor model construction and irradiation in a mouse tumor model. **B** Tumor growth curves of unirradiated (Control), RTRT, and CNRT in a mouse tumor model (n = 5). ^#^, Control vs CNRT. ^*^, Control vs RTRT. ^#^/^*^, *P* < 0.05. ^###^/^***^, *P* < 0.001. ^####^/^****^, *P* < 0.0001. The statistical test was performed using One-way ANOVA, followed by Tukey’s multiple comparisons test. **C** Tumor growth curves for each mouse in control and RTRT groups. **D** Tumor growth curves for each mouse in control and CNRT groups. **E** Kaplan–Meier survival curves were generated to compare mortality between groups, and significance was determined by log-rank (Mantel-Cox) test. ^*^, *P* < 0.05. **F**, **G** Physical map of lung tissue and quantification of lung metastasis of tumors after control, RTRT, and CNRT in a mouse tumor model. H, I Immunohistochemical images and quantification of Ki67^+^ cells in each group. ^*^, *P* < 0.05. ns indicates no statistical difference. The statistical test was performed using One-way ANOVA, followed by Tukey’s multiple comparisons test

To assess the anti-tumor effects of RTRT, we employed two irradiation modalities and compared their impact on tumor growth. Both treatments significantly delayed tumor progression compared to the control. The tumors growth was significantly inhibited about one week post-irradiation, and with RTRT exhibiting comparable anti-tumor effects to CNRT (Fig. 2B). Irradiation effectively inhibited tumor lung metastasis and prolonged survival time, with RTRT showing a superior survival benefit relative to control (Fig. 2E–G). IHC analysis revealed a significant reduction in Ki67^+^ proliferating tumor cells following both treatments, with no difference between RTRT and CNRT, indicating that both therapies effectively inhibit tumor cell proliferation (Fig. 2H, I). These results suggest that RTRT, at approximately 40% of maximum cross-sectional area, has a similar anti-tumor efficacy with CNRT.

### Normal tissue-protective effects of RTRT

**Fig. 3:**
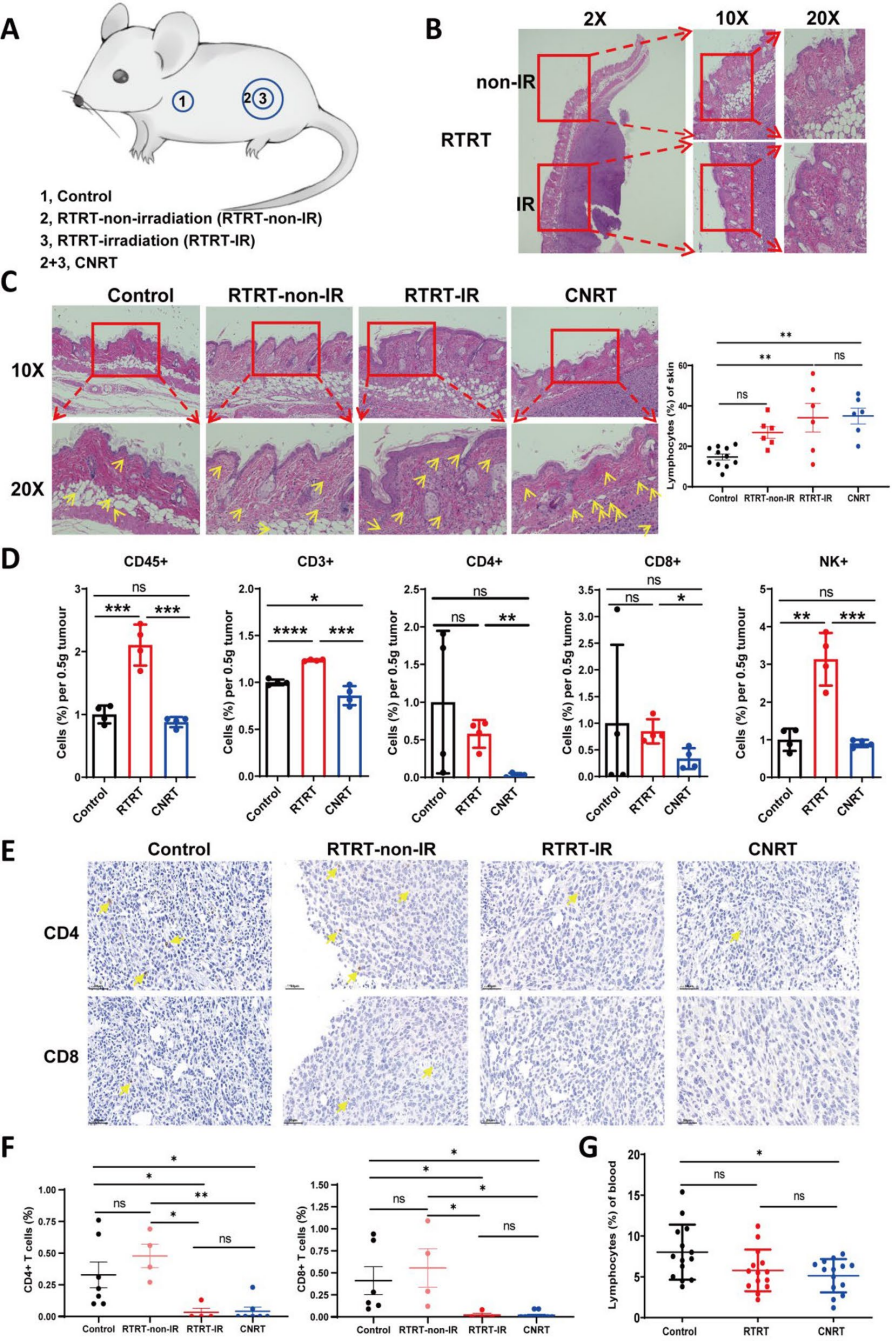
RTRT protects skin and lymphocytes. **A** Schematic representation of different treatments of mouse skin tissues. **B** Representative picture of HE staining of skin lesions on the surface of tumors treated with RTRT. **C** HE staining images of skin tissue and skin infiltrating lymphocytes of mice in control, RTRT and CNRT group (Left). Quantification of skin infiltrating lymphocytes in each group (Right). Yellow arrows indicate lymphocytes infiltration. **D** Analysis of immune cell subsets in tumor tissues of each group by Flow cytometric analysis. n = 4. **E** Immunohistochemical images of CD4^+^ and CD8^+^ T cells in tumor of control, RTRT and CNRT group. Yellow arrows indicate lymphocytes infiltration. **F** The quantification of CD4^+^ and CD8^+^ T cells in tumor of each group. **G** Percentages of lymphocytes in peripheral blood of mice irradiated with different methods. ^*^, *P* < 0.05. ^**^, *P* < 0.01. ^***^, *P* < 0.001. ns indicates no statistical difference. The statistical test was performed using One-way ANOVA for multiple groups, followed by Tukey’s multiple comparisons test. The statistical test was performed using Student’s t-test for two groups

To further determine the effects of RTRT and CNRT on normal tissues, we analyzed skin and peripheral blood samples from mice treated with two treatments. Skin damage at the irradiated sites was similar between the two irradiation methods. We observed mild thickening of the epidermal surface and structural disruptions in the stratum corneum and epidermis, including disorganization, an increase in lymphocyte infiltration, and vascular structural changes (Fig. 3B, C; Fig. S1). The unirradiated skin in the RTRT group showed no significant histological differences compared to the control group, and RTRT had a tendency to promote the increase of lymphocytes in surrounding normal skin (Fig. 3C). These results suggest that RTRT may offer protects to adjacent normal tissue while enhancing immune cell infiltration, thereby potentially boosting the local immune response. Peripheral blood analysis showed no significant change in lymphocyte percentages between RTRT and control groups, whereas a significant reduction was observed in CNRT (Fig. 3G), further indicating that RTRT somewhat protected lymphocytes from radiation damage.

### RTRT preserved tumor-infiltrating lymphocytes

Next, we analyzed the percentage of lymphocytes in tumor by flow cytometry and IHC (Fig. 3D–F). Our results showed a significant increase in the proportion of lymphocytes within the tumor following RTRT, particularly NK cells, when compared to the control group. This increase was not observed in the CNRT-treated tumors (Fig. 3D). Moreover, RTRT treatment resulted in a notable elevation of CD4^+^, CD8^+^ T cells, and NK cells within the tumor microenvironment, in contrast to CNRT (Fig. 3D). IHC analysis revealed a significant reduction in the presence of CD4^+^ and CD8^+^ T cells in irradiated tumors, while the lymphocyte population in non-irradiated portion (RTRT-non-IR) did not change (Fig. 3E, F). The above results suggest that CNRT kills tumor-infiltrating lymphocytes along with tumor cells, whereas RTRT preserves the lymphocyte population surrounding the irradiated tumor.

### Partial irradiation of tumor cells could induce DC maturation

**Fig. 4:**
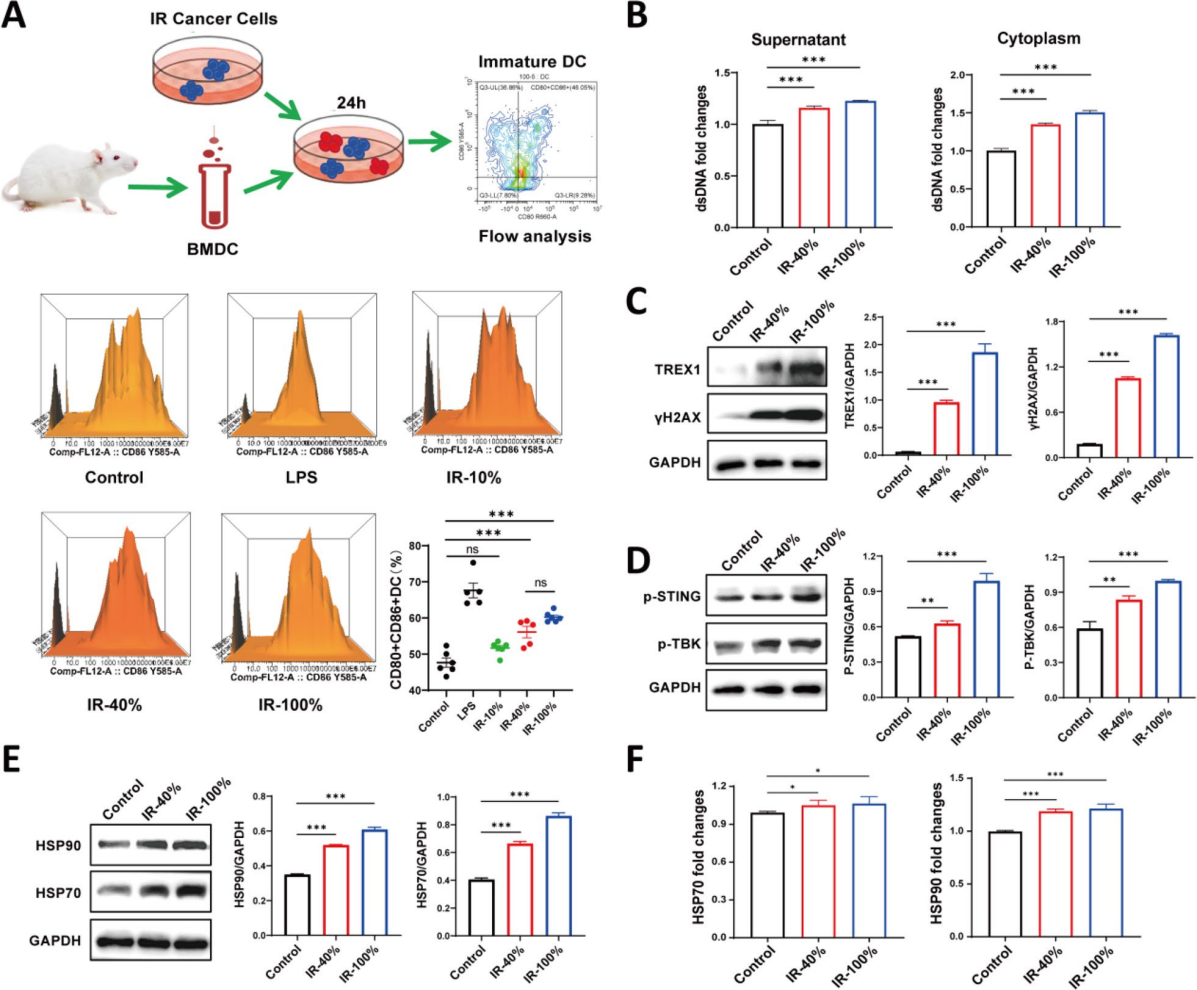
DNA damage plays an important role in radiation-induced immune activation. **A** Schematic representation of irradiated tumor cells inducing maturation of murine bone marrow-derived DCs. Flow peak of a proportion of CD86^+^ CD80^+^ DC in each group. The proportion of CD86^+^ CD80^+^ DC in each group. Flow cytometric analysis of CD86^+^ CD80^+^ DC in each group. n = 6. **B** Levels of dsDNA in culture supernatant and cytoplasm of tumor cells in each group. **C** Expression and quantification of DNA damage and repair proteins. **D** Expression and quantification of cGAS-STING pathway proteins. **E** Expression and quantification of DAMPs. **F** Relative expression levels of HSP70 and HSP90 in the supernatants after different treatments. ns indicates no statistical difference. ^*^, *P* < 0.05. ^**^, *P* < 0.01. ^***^, *P* < 0.001. The statistical test was performed using One-way ANOVA, followed by Tukey’s multiple comparisons test

We next examined the immunogenicity of tumors subjected to different irradiation ratios by assessing dendritic cell (DC) maturation. Bone marrow-derived DC cells (BMDC) were co-cultured with different percentages of irradiated tumor cells, and DC maturation was evaluated by flow cytometry. Lipopolysaccharide (LPS) treatment was used as a positive control group. Our results revealed that 40% and 100% irradiated tumor cells increased the proportion of CD80^+^ CD86^+^ DCs compared to the control (Fig. 4A). In contrast, 10% irradiated tumor cells do not significant induce maturation of DCs. Moreover, 40% of irradiated tumor cells achieve a percentage of mature DCs, which was not significantly different from that of IR-100% (Fig. 4A), suggesting that a partial percentage of irradiated tumor cells also effectively promote DC maturation.

### RT-induced DNA damage/DAMPs and activated cGAS-STING signaling

To understand the DNA damage in irradiated tumor cells with different ratios, we examined the release of dsDNA and associated markers in vitro. Compared to control conditions, tumor cells subjected to IR-40%, IR-50%, and IR-100% showed a significantly increase in dsDNA release both supernatant and cytoplasm (Fig. 4B, Fig. S3A). Meanwhile, compared to the control, we observed elevated expression of DNA damage proteins γH2AX and DNA damage repair protein TREX1 following irradiation, the most pronounced effects seen at IR-100% (Fig. 4C). Subsequently, we found that dsDNA released from partially irradiated tumor cells increased the level of phosphorylation STING and activated downstream TBK protein, although the response was less pronounced compared to 100% irradiation (Fig. 4D, Fig. S3C). DAMPs act as danger signals to trigger anti-tumor immune responses together with tumor antigens. We observed the HSP70 and HSP90 expression was elevated in irradiated tumor cells compared to unirradiated ones (Fig. 4E, F; Fig. S3D, E). Irradiation of 50% of tumor cells induced similar levels of DAMPs release, DNA damage, and cGAS-STING pathway activation as observed with 40% irradiation, with a higher trend (Fig. S3B-E). These data suggest that partial tumor cells irradiation may promote the release of dsDNA, DAMPs, and activation of the STING pathway, thereby contributing to an enhanced immune response.

### ***CD4***^+^***T/CD8***^+^***T/NK cells played different roles in the anti-tumor effects of RTRT and CNRT***

**Fig. 5:**
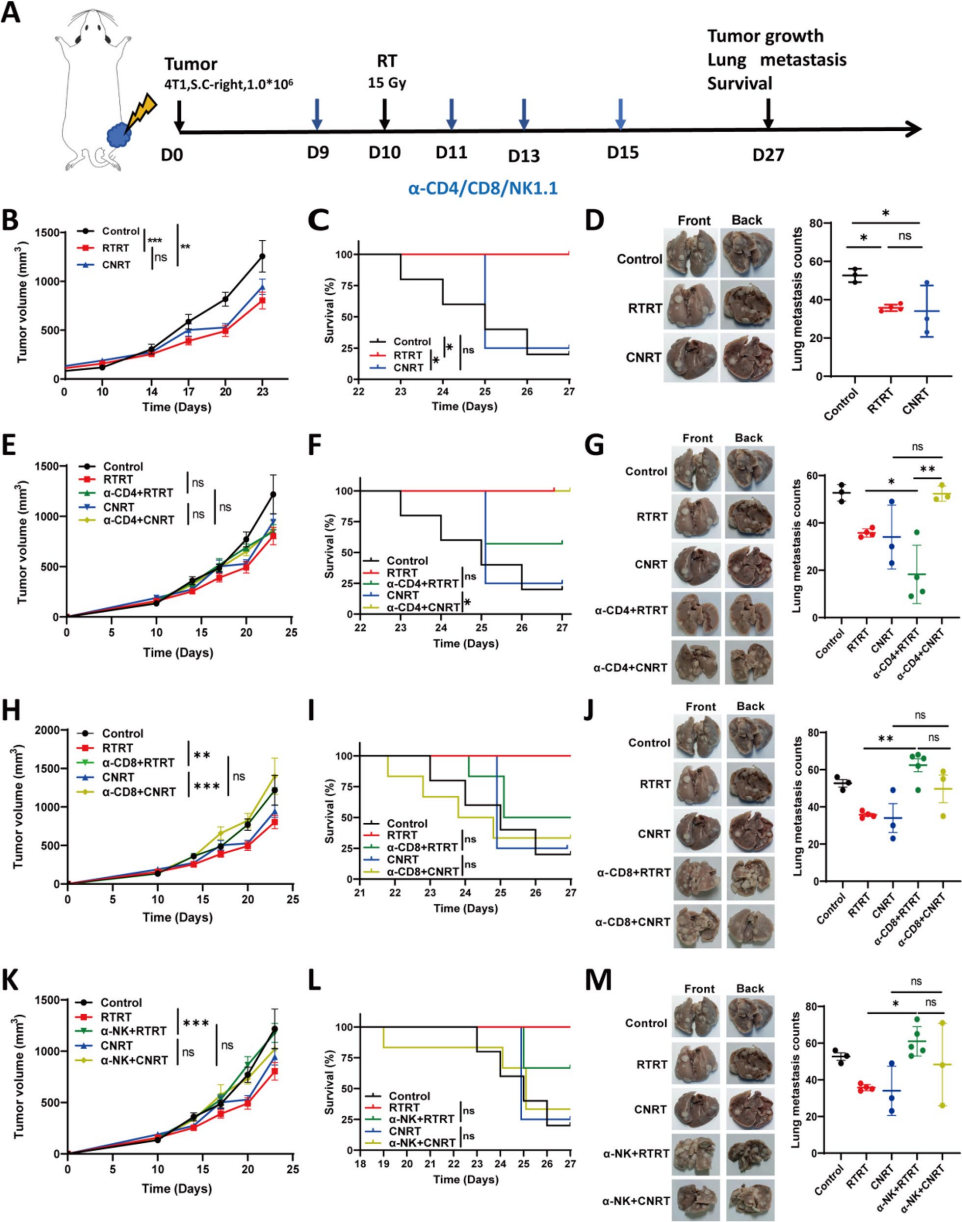
CD4^+^ T/CD8^+^ T/NK cells is involved in the anti-tumor effect of RTRT and CNRT. **A** Schematic diagram of CD4^+^ T/CD8^+^ T/NK cells antagonist mouse model construction. **B**, **E**, **H**, **K** tumor growth curves of control, RTRT, and CNRT of mice with or without antagonistic CD4^+^ T/CD8^+^ T/NK cells. n ≥ 4. ^*^, *P* < 0.05. ^**^, *P* < 0.01. ^***^, *P* < 0.001. ns indicates no statistical difference. The statistical test was performed using Two-way ANOVA, followed by Tukey's multiple comparisons test. **C**, **F**, **I**, **L** Kaplan–Meier survivalcurves were generated of control, RTRT, and RTRT of mice with or without antagonistic CD4^+^ T/CD8^+^ T/NK cells, and significance was determined by log-rank (Mantel-Cox) test. ^*^P < 0.05. **D**, **G**, **J**, **M** Physical images of lung metastases in each group of mice in the CD4^+^ T/CD8^+^ T/NK cells antagonist model (Left). Quantitative analysis of lung metastasis in each group of mice in the CD4^+^ T/CD8^+^ T/NK cells antagonistic model (Right). n ≥ 3. ^*^, *P* < 0.05. ^**^, *P* < 0.01. ^***^, *P* < 0.001. ns indicates no statistical difference. The statistical test was performed using One-way ANOVA, followed by Tukey's multiple comparisons test

Next, we used CD4^+^ T/CD8^+^ T/NK cell antibodies to antagonize the corresponding lymphocyte subsets before radiotherapy to evaluate the contribution of different immune cells to the anti-tumor effects of RT (Fig. 5A). The results again demonstrated that RTRT exhibited a similar anti-tumor effect to CNRT in inhibiting tumor growth and lung metastasis (Fig. 5B, D). Moreover, RTRT has a superior survival benefits compared to CNRT (Fig. 5C). However, the tumor growth inhibition of RTRT was attenuated upon depletion of the corresponding immune cell populations with the pronounced reduction observed following depletion of CD8^+^ T and NK cells (Fig. 5H, K). In CNRT, tumor inhibition was also attenuated after depletion of CD8^+^ T cells, but the depletion of CD4^+^ T and NK cells did not significantly affect radiation-induced tumor suppression (Fig. 5E, H, K). The above results suggest that CD8^+^ T cells play a dominant role in mediating the tumor-suppressive effects of both RTRT and CNRT.

Survival curves further elucidated the impact of immune cell depletion on the therapeutic efficacy of RT. Depletion of CD4^+^ T, CD8^+^ T, or NK cells in the RTRT group did not significantly attenuate the radiation-induced survival benefit, although it reduced survival at the study endpoint (Fig. 5F, I, L). Similarly, depletion of CD8 and NK in CNRT did not significantly affect the overall survival time (Fig. 5I, L). Interestingly, anti-CD4 in CNRT unexpectedly prolonged survival (Fig. 5F), which might be related to the incitement of TGF-β transcription after antagonising CD4^+^ T cells (Fonseca et al. 2022). These results suggest that CD4^+^ T cells, CD8^+^ T cells, and NK cells all contribute to the radiation-induced prolongation of survival, though CD4^+^ T cells seem to be appear to be detrimental in the context of CNRT.

We simultaneously detected lung metastasis in all groups. In the RTRT model, depletion of CD8^+^ T and NK cells resulted in a increase in tumor metastasis compared to RTRT alone. In CNRT mice, there was a tendency for CD4^+^ T, CD8^+^ T, and NK cell depletion to increase lung metastasis, but the difference was not statistically significant (Fig. 5G, J, M). This suggests that CD8^+^ T and NK cells contribute to the reduction of lung metastasis in RTRT.

Collectively, These results imply that CD4^+^ T/CD8^+^ T/NK cells play an important role in the anti-tumor effect of radiation, but the role of individual cell subpopulations in two irradiation modes is different.

### RTRT induced abscopal effect to control distal tumors

**Fig. 6:**
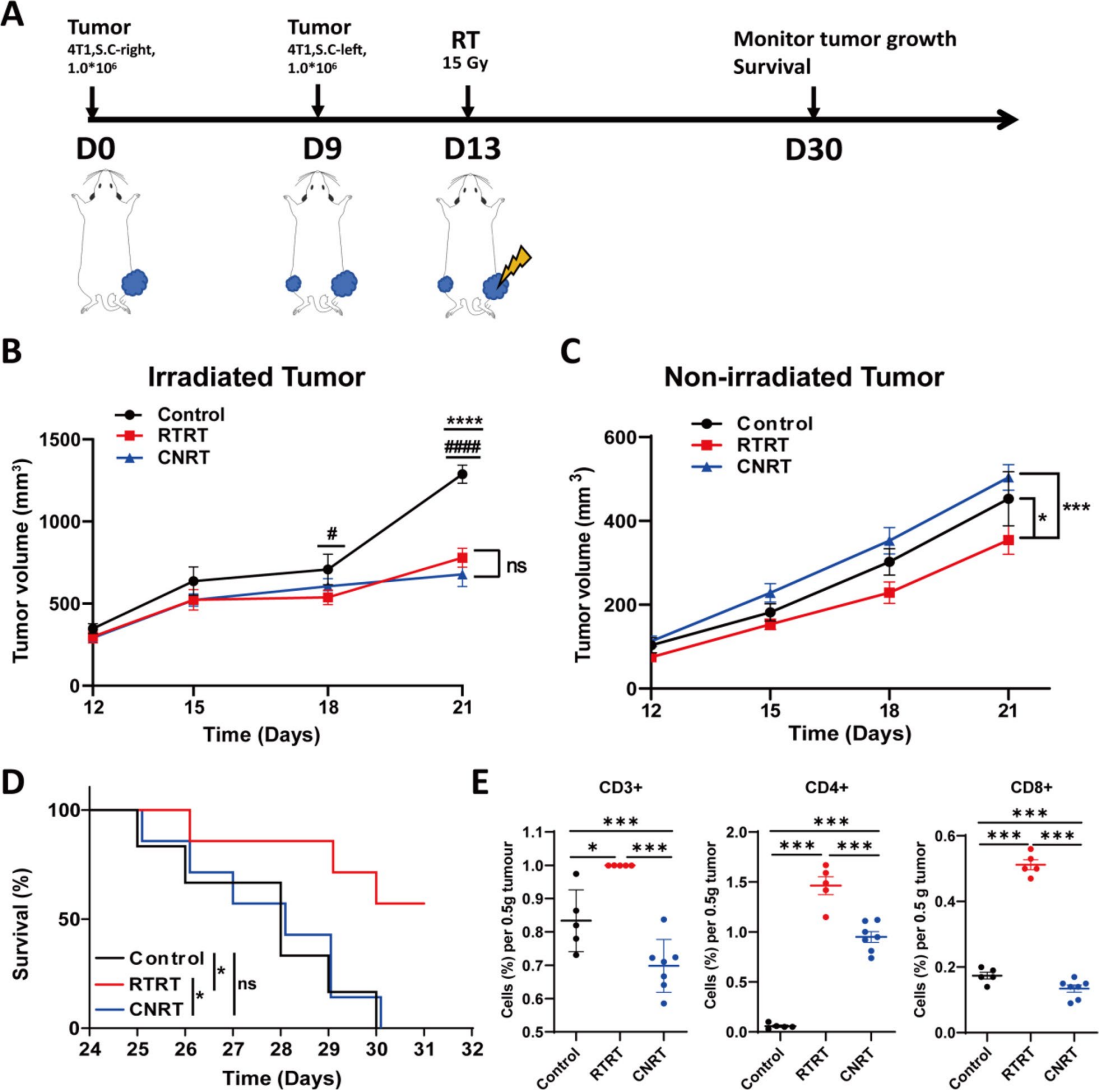
The ability of RTRT and CNRT in eliciting abscopal effects. **A** Schematic of bilateral tumor model construction and irradiation. **B** Growth curves of irradiated tumors in a bilateral tumor model (n = 5). ^#^, RTRT vs Control, *P* < 0.05. ^####^, RTRT vs Control, *P* < 0.0001. ^****^, CNRT vs Control, *P* < 0.0001. ns indicates no statistical difference. The statistical test was performed using One-way or Two-way ANOVA, followed by Tukey’s multiple comparisons test. **C** Growth curves of non-irradiated tumors in a bilateral tumor model. ^#^, RTRT vs Control, *P* < 0.05. ^***^, CNRT vs Control, *P* < 0.001. The statistical test was performed using Two-way ANOVA, followed by Tukey's multiple comparisons test. **D** Kaplan–Meier survival curves were generated of each group mice in the bilateral tumor model, and significance was determined by log-rank (Mantel-Cox) test. ^*^, *P* < 0.05. **E** Lymphocyte subsets of non-irradiated tumors were analyzed by flow cytometry (n ≥ 5). ns indicates no statistical difference. ^*^, *P* < 0.05. ^**^, *P* < 0.01. ^***^, *P* < 0.001. The statistical test was performed using One-way ANOVA, followed by Tukey’s multiple comparisons test

RT has been shown to exert abscopal effects, whereby local irradiation can suppress the growth of distal, non-irradiated tumors through systemic immune activation. To explore the similarities and differences of RTRT and CNRT on inducing abscopal effect, we irradiated the larger tumor in bilaterally loaded tumors mice and monitored the growth of both irradiated and distal tumors, respectively (Fig. 6A). Both irradiation methods significantly inhibited tumor growth in situ (Fig. 6B). However, only RTRT prolonged the survival of the mice, while CNRT group mice did not gain the survival benefit (Fig. 6D). Amazingly, RTRT induced a abscopal effect, inhibiting the growth of non-irradiated distal tumors, whereas CNRT did not (Fig. 6C). Next, flowcytometric analysis revealed a significant increase in tumor-infiltrating lymphocytes (TILs) in non-irradiated tumors in the RTRT-treated group, such as CD4^+^ and CD8^+^ T cells (Fig. 6E). This finding suggests that the increase of lymphocytes in non-irradiated tumor tissues induced by RTRT may contribute to the enhanced abscopal effect observed in this treatment regimen.

## Discussion

Radiotherapy is considered one of the most cost-effective modalities for cancer treatment and is commonly used for clinical tumor control. However, to achieve effective tumor suppression, high doses of radiation are necessary to overcome radioresistant tumor cells. A challenge arises from the differing radiation tolerance between normal tissues and tumor tissues, leading to potential damage to normal tissues, particularly radiosensitive immune cells. Novel radiotherapy techniques, such as FLASH (Zhang et al. 2021; Wardman 2023) and proton radiotherapy (Ramella and D’Angelillo 2020; Yan et al. 2023), have demonstrated effective tumor control through ultra-high dose rates and Bragg-peak distributions, respectively. Nonetheless, the limited availability and high costs of these advanced treatments restrict patient accessibility, failing to meet clinical demands for cancer therapy. Small-field radiotherapy has shown promising anti-tumor potential in some studies and patient cohorts (Vicini et al. 2003; Conway et al. 2017). In the present study, we investigated the balance between tumor control and normal tissue protection in radiotherapy and found that irradiating approximately 40% of the tumor tissue achieved anti-tumor effects comparable to CNRT, while simultaneously reducing normal tissue toxicity. This finding aligns with previous studies, such as Frank’s (Vicini et al. 2003), which report that limited-field radiotherapy is similar efficacy in breast cancer patients compared to whole-breast radiotherapy. Additionally, Tubi et al. demonstrated that SBRT-PATHY can enhance the antitumor effect of radiotherapy by preserving peritumoral immune cells (Tubin et al. 2020). In patients with advanced squamous cell carcinoma of the neck, a single dose of 20 Gy GRID followed by chemotherapy achieved an overall tumor control rate of 79%, with tolerability similar to that of simultaneous integrated boost intensity-modulated radiotherapy (SIB-IMRT), and acute toxicity similar to chemotherapy alone (Penagaricano et al. 2010). Lattice radiotherapy (LRT), based on GRID technology, significantly improved local control rates in non-small cell lung cancer patients with mild adverse effects (Amendola et al. 2018). These results suggest that appropriately reducing the irradiation field can not only maintain therapeutic efficacy but also alleviate radiation-induced toxicity.

Metastasis is a leading cause of cancer-related deaths (Ganesh and Massague 2021; Gerstberger et al. 2023). Most cancer patients are diagnosed with metastases, which presents both challenges and focal points in treatment strategies. Both CNRT and RTRT inhibit tumor metastasis compared to the untreated group, with no difference between them (Fig. 2F, G). Beyond tumor cell killing, radiation therapy also induces an abscopal effect-a rare phenomenon where unirradiated tumors are suppressed (Abuodeh et al. 2016; Janopaul-Naylor et al. 2021). In bilaterally loaded mice, we observed that RTRT is more likely to induce an abscopal effect, suppress distant tumors and improving survival outcomes (Fig. 6A–D). This may be associated with a greater retention of peripheral tumor-infiltrating lymphocytes (Fig. 6E), a finding consistent with the SBRT-PATHY study by Tubi et al. (2020).

Tumor radiotherapy often results in damage to adjacent organs or normal tissues, which not only limits the ability to escalate radiation doses but also reduces the overall effectiveness of the treatment (Seo et al. 2019). Tumor cells, due to their proliferative demands, induce significant neoangiogenesis within tumor tissue (Tozer et al. 2005; Geindreau et al. 2022). Immune cells of blood are particularly sensitive to radiation, with extremely low doses (10–25 rad) of X-rays causing cytotoxic T cells death in mice (Spellman and Anderson 1982). Naïve T cells and hematopoietic stem cells undergo rapid apoptosis (within hours) after exposure to low-dose irradiation (Gudkov and Komarova 2003). CNRT typically uses fractionated treatment with a single dose around 2 Gy, with total doses up to 70 Gy, targeting both the entire tumor tissue and a 5 mm margin of surrounding tissue (Zietman et al. 2005). While effective at killing tumor cells, CNRT also causes damage to infiltrating lymphocytes and adjacent healthy tissues. Approximately 70% of patients undergoing clinical radiotherapy develop radiation-induced lymphopenia (RIL) syndrome (Afanasiev et al. 2013; Balermpas et al. 2016; Horn et al. 2017), which contributes to reduced survival rates in patients with malignant glioma, lung, and pancreatic cancer (Horn et al. 2017). In contrast, peripheral blood lymphocyte analysis in mice revealed a higher proportion of lymphocytes following RTRT treatment (Fig. 3G), indicating that RTRT preserves some lymphocytes in the peripheral blood, which supports the activation of the immune response. In contrast, CNRT’s significant reduction in circulating immune cells may impair immune defenses.

By killing immune cells within the tumor, CNRT essentially converts immunologically “hot” tumors into “cold” tumors. Despite the release of more tumor-associated antigens and cytokines, adaptive immune responses are weak due to the lack of infiltrating cytotoxic lymphocytes. Immune microenvironment analysis showed that, compared to CNRT, RTRT more effectively preserved lymphocytes within the tumor, particularly CD4^+^ T and CD8^+^ T cells (Fig. 3D, F), and maintained the tumor immune microenvironment. These findings suggest that RTRT may offer greater potential for combining with immunotherapy in cancer treatment.

In vitro, co-culturing experiments with irradiated cells and BMDCs revealed that varying proportions of irradiated tumors cells may induce DC maturation. Particularly, at moderate proportions (about 40%), a higher the proportion of irradiated tumor cells, the stronger the induction of DC maturation. However, beyond a certain proportion (threshold), further increasing the proportion of irradiated tumor cells did not result in a greater proportion of mature DCs (Fig. 4A). These data indicate that above a moderate proportion of irradiated tumor cells could efficiently induce BMDC maturation.

Radiation-induced DNA damage and new antigens are crucial for systemic tumor regression (Lippert and Greenberg 2021). The dsDNA induced by radiation is a key factor in tumor tissue destruction and serves as the “trigger” for subsequent anti-tumor immune responses. cGAS, combined with dsDNA from dying tumor cells, catalyzes the production of the second messenger cyclic GMP-AMP synthase (cGAMP). This activates the stimulator of interferon genes (STING) protein, recruits the serine-threonine protein kinase TBK1, which in turn activates IRF3, leading to the production of type I interferons and immune factors (Mackenzie et al. 2017). Although RTRT induced cytoplasmic dsDNA is not as strong as CNRT, it is sufficient to activate the cGAS-STING pathway and promote immune activation. When dsDNA occurs, H2AX accumulates at the DNA breakage site and is rapidly phosphorylated by members of the phosphoinositide 3-kinase-related kinase (PIKK) family, including ataxia-telangiectasia mutated (ATM), ataxia-telangiectasia and Rad3-related (ATR), and DNA-activated protein kinase (DNA-PK), forming phosphorylated histone H2AX (γH2AX), a well-established marker of DNA damage (Harper and Elledge 2007; Rothkamm et al. 2007; Beels et al. 2009). The DNA exonuclease TREX1 plays a critical role in regulating the immune balance of cGAS signaling and acts as an upstream modulator of radiation-induced anti-tumor immunity. TREX1 expression is dose-dependent. At higher radiation doses (12 Gy and 18 Gy), TREX1 is sufficient to scavenge cytoplasmic dsDNA accumulated in some cancer cells, thus preventing excessive activation of the cGAS-STING pathway and inhibiting the downstream interferon (IFN-I) responses (Cai et al. 2014; Vanpouille-Box et al. 2017). Compared to non-irradiated tumor cells, TREX1 and γ-H2AX expression levels were elevated in tumor cells subjected to partial or whole irradiation at 12 Gy, indicating that while TREX1 expression in 4T1 tumor cells irradiated at 12 Gy can reduce cytoplasmic dsDNA accumulation, it does not fully degrade the dsDNA or prevent STING activation. This hypothesis was further supported by the increase expression of p-STING following both partial and whole-tumor irradiation (Fig. 4B–D, Fig. S3A–C).

In conclusion, inspired by small field irradiation, we explored the relationship between tumor control and immune preservation mediated by reducing the tumor radiotherapy target volume, and revealed that RTRT has a comparable tumor control and metastasis inhibition effect to CNRT in various preclinical models, while providing better survival benefits. This improvement is primarily due to the retention of tumor-associated immune cells within tumor and blood following RTRT treatment. Furthermore, RTRT is more likely to induce an abscopal effect, effectively suppressing distant tumor growth, and thereby improving antitumor efficacy. Mechanistically, RTRT induced catastrophic dsDNA in tumor cells, which activated the cGAS-STING pathway. The concurrent release of tumor antigens and DAMPs synergistically enhanced anti-tumor immune responses. These findings provide a possible solution for optimizing traditional radiotherapy target areas, and thereby reducing radiation damage to normal tissues. Moreover, RTRT preserved and enhanced infiltrating lymphocytes within the tumor, offering the possibility of combining radiotherapy with immunotherapy to strengthen the anti-tumor response. However, there are several limitations to our study. First, the types of tumors investigated in this study are limited, and the antitumor effects of RTRT in other cancer types remain unclear. Second, only a single irradiation parameter was used in this study, which may not fully capture the anti-tumor effect of RTRT’s under different treatment conditions. Future studies should explore the effects of partial irradiation at different clinical radiotherapy doses across a broader range of tumor types, which will provide further insights into the optimization of tumor radiotherapy. In short, reducing the irradiated area improved tumor treatment efficacy, mitigated side effects, and preserved lymphocytes, which may increase the incidence of abscopal effects. This approach has the potential to benefit cancer patients who require high-dose irradiation, improving both the efficacy and safety of radiotherapy.

## Supplementary Information

Supplementary Material 1.

## Data Availability

The data that support the findings of this study are available from the corresponding authors upon reasonable request.
