# Correction: Reduced irradiation exposure areas enhanced anti-tumor effect by inducing DNA damage and preserving lymphocytes

**DOI:** 10.1186/s10020-025-01382-4

**Published:** 2025-11-03

**Authors:** Huiqin Chen, Yuan Li, Qiaofeng Shen, Guanqun Guo, Zhigang Wang, Hanyu Pan, Min Wu, Xueqing Yan, Gen Yang

**Affiliations:** 1 State Key Laboratory of Nuclear Physics and Technology, School of Physics, Peking University, Beijing, 100871 China; 2 Oncology Discipline Group, the Second Affiliated Hospital of Wenzhou Medical University, Wenzhou, 325003 China; 3 Wenzhou Institute, University of Chinese Academy of Sciences, Wenzhou, 325000 China; 4 School of Public Health, Wenzhou Medical University, Wenzhou, 325035 China; 5 South Zhejiang Institute of Radiation Medicine and Nuclear Technology, Wenzhou, 325014 China

**Correction: Molecular Medicine 30, 284 (2024)**

10.1186/s10020-024-01037-w

In this article [1], there is a duplication of the funding information in both “the Acknowledgements section” and “the Funding section”.

The Acknowledgements section of this article [1], should have been “We hereby desire to express indebtedness to anonymous reviewers for their valuable and constructive comments. We would like to acknowledge Dr. Xiaoji Lin for his valuable contributions to the animal irradiation experiments.”

The affiliation details for Author Xueqing Yan were incorrectly given as “Oncology Discipline Group, the Second Affiliated Hospital of Wenzhou Medical University, Wenzhou 325003, China” but should have been “State Key Laboratory of Nuclear Physics and Technology, School of Physics, Peking.

University, Beijing 100871, China”.

Affiliation 5 “Oncology Discipline Group, the Second Affiliated Hospital of Wenzhou Medical University, Wenzhou 325003, China” was mistakenly included in the affiliation list which has been deleted and renumbered the affiliation list.

The original article has been corrected.
