# Supplementary material for: Reduced irradiation exposure areas enhanced anti-tumor effect by inducing DNA damage and preserving lymphocytes
Source: Mol Med. 2024 Dec 31;30:284. doi: 10.1186/s10020-024-01037-w (PMC11687019; doi:10.1186/s10020-024-01037-w)
Supplement: Supplementary file 1 — Supplementary Material 1. [file 10020_2024_1037_MOESM1_ESM.docx]

**Supplementary Figures**

**
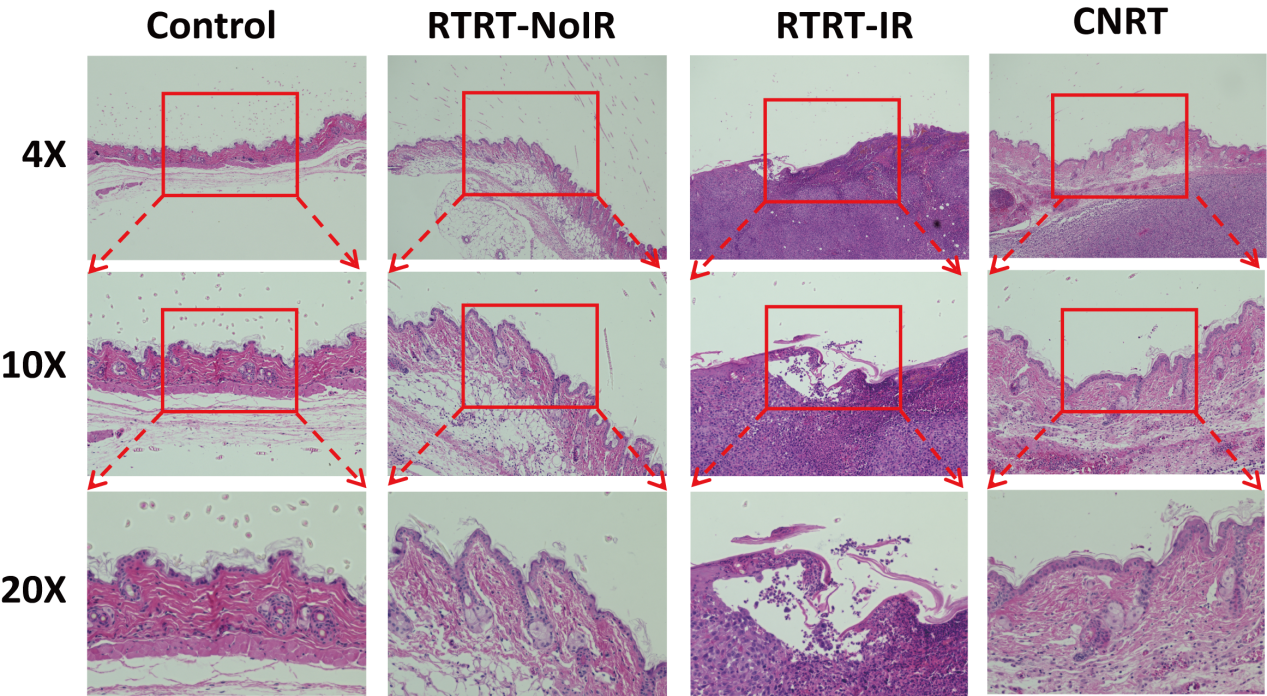
**

**Fig. S1 The skin of different treatments was stained with HE.** HE images of mice skin in different treatment groups under different magnification.


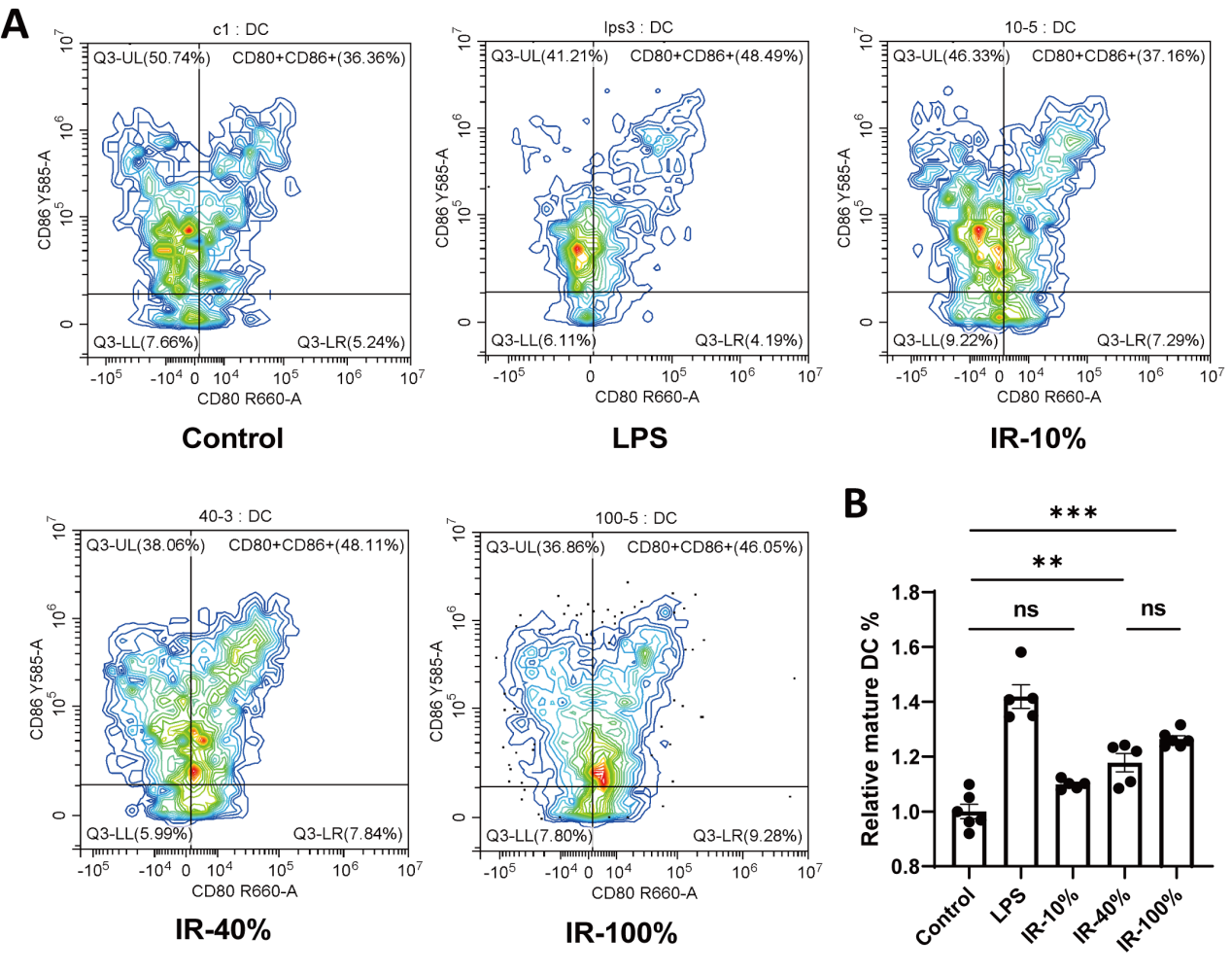


**Fig. S2 Percentage of mature DCs induced by co-culture of irradiated tumor cells and BMDC in vitro.** **A,** Representative plot of CD80+CD86+ double positive DC in each group. **B,** Relative percentage of mature DC in each group. **，*P*<0.01. ***，*P*<0.001. The statistical test was performed using One-way ANOVA, followed by Tukey's multiple comparisons test.


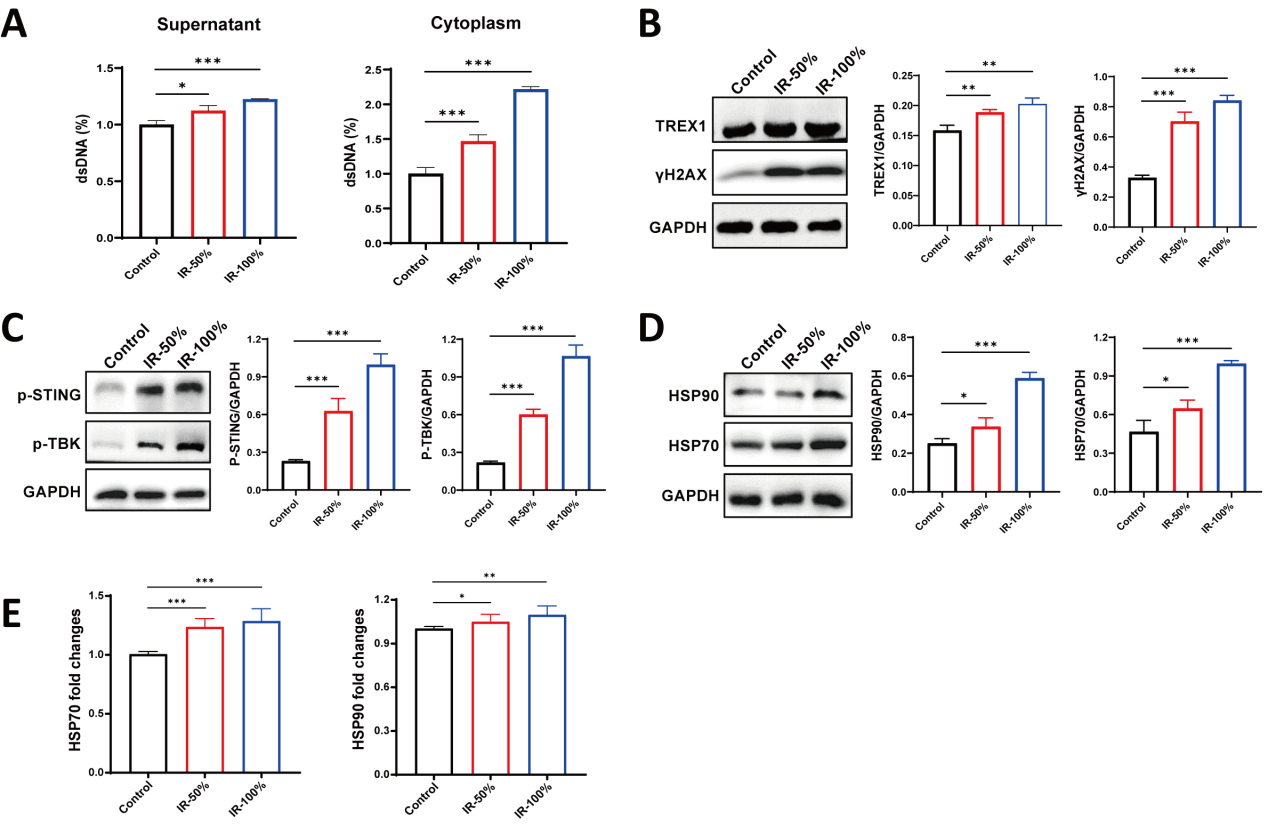


**Fig. S3 50%-IR induced dsDNA/DAMPs and cGAS-STING activation.** **A,** Levels of supernatant, cytoplasmic and nuclear dsDNA in tumor cells of each group. B, Expression and quantification of DNA damage and repair proteins. **C,** Expression and quantification of cGAS-STING pathway proteins. **D,** Expression and quantification of DAMPs. **E,** Relative expression levels of HSP70 and HSP90 in supernatants after different treatments. *，*P*<0.05. **. *P*<0.01. ***，*P*<0.001. ****，*P*<0.0001. The statistical test was performed using One-way ANOVA, followed by Tukey's multiple comparisons test.


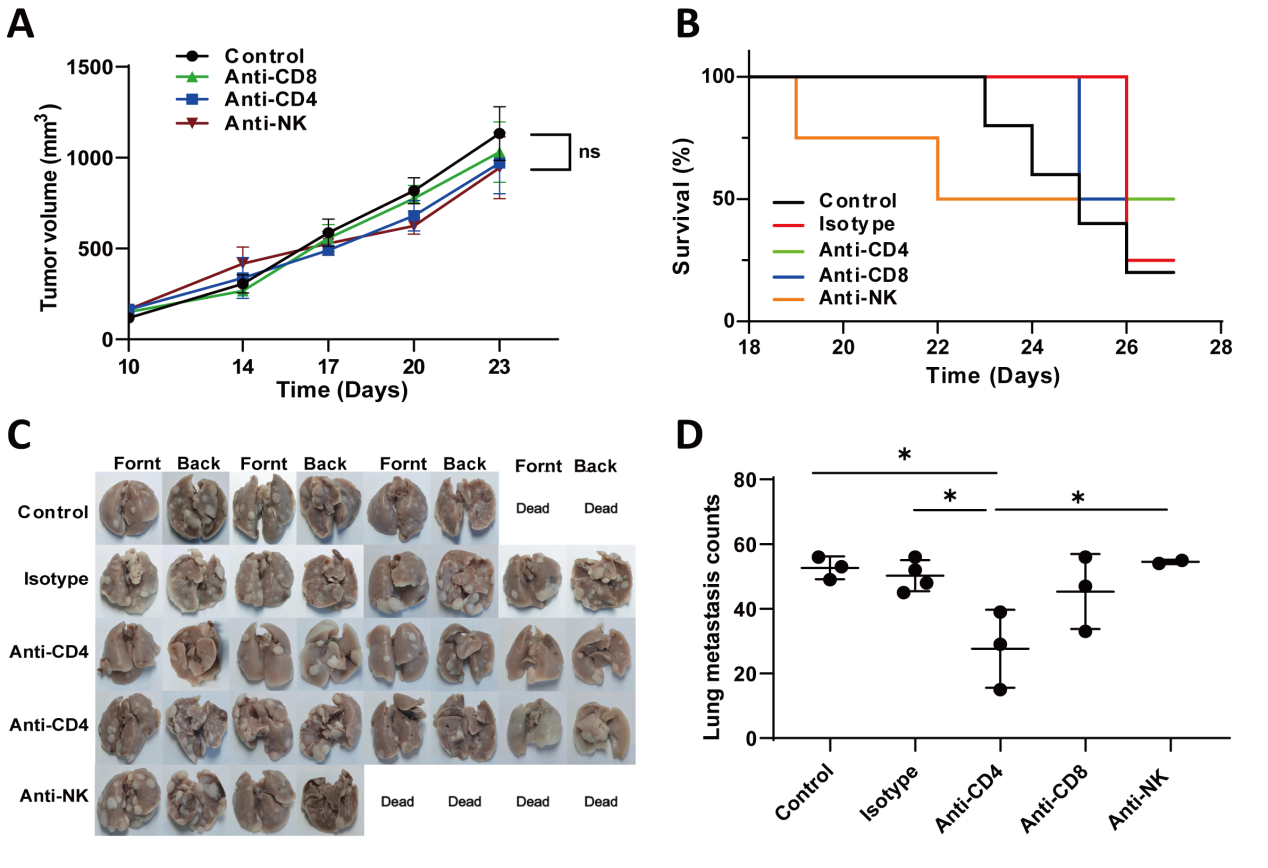


**Fig. S4 Lung metastasis and CD4/CD8/NK cells mono-antagonistic effect.** **A,** tumor growth curves in the CD4/CD8/NK cells antagonist alone group. **B,** Kaplan-Meier survivalcurves were generated to compare mortality in the CD4/CD8/NK cells antagonist alone group. **C,** Physical images of lung metastases in mice with single CD4/CD8/NK cells antagonism. **D,** Quantitative analysis of lung metastasis in the CD4/CD8/NK cells antagonist alone group. ns indicates no statistical difference. *, *P*<0.05. The statistical test was performed using One-way ANOVA, followed by Tukey's multiple comparisons test.
